# Supplementary figures and images for: Seipin traps triacylglycerols to facilitate their nanoscale clustering in the endoplasmic reticulum membrane
Source: PLoS Biol. 2021 Jan 22;19(1):e3000998. doi: 10.1371/journal.pbio.3000998 (PMC7857593; doi:10.1371/journal.pbio.3000998)

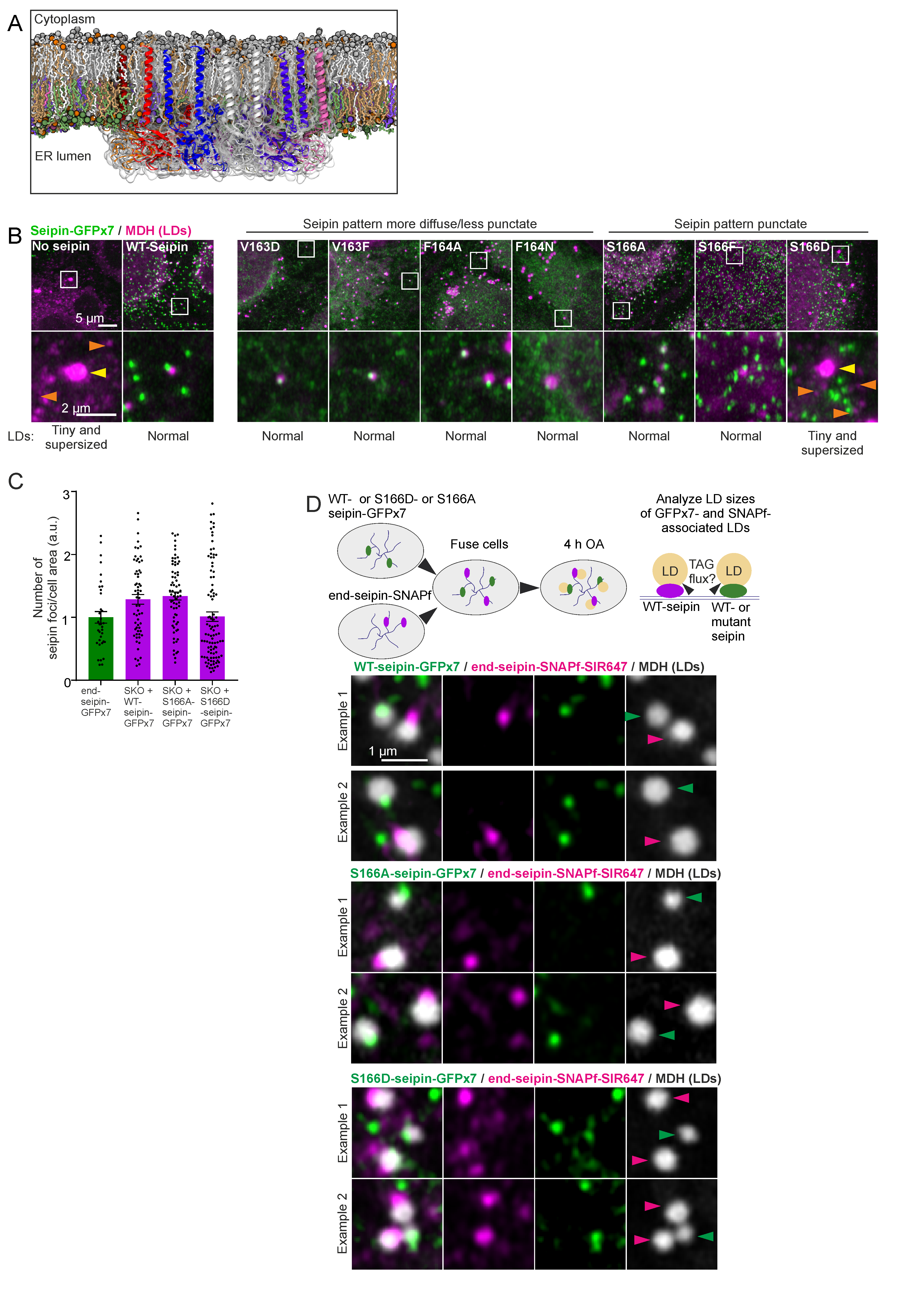

Supplement: S1 Fig — (A) Side view of the seipin oligomer in the model ER bilayer. The model was based on the cryo-EM structure of human seipin, and the TMDs were modeled. The lipid composition was modeled to match that reported for the ER. (B) A431 SKO cells stably expressing the indicated plasmids were delipidated for 3 d and treated with 200 μM OA for 1 h. Cells were fixed, LDs stained, and cells imaged by Airyscan microscopy. Orange arrowheads: tiny LDs; yellow arrowheads: supersized LDs. (C) Related to Fig 1E and 1F. Indicated cell lines were delipidated for 3 d and treated with OA for 1 h. Cells were fixed, LDs stained, and cells imaged by Airyscan microscopy. The number of seipin foci per cell area was analyzed and plotted. n = 35 cells for end-seipin-GFPx7, >60 cells/group for the others, 3–4 experiments. (D) Exemplary crops of nearby LDs from cells treated as indicated. Green arrowheads: WT-, S166A-, or S166D-seipin-GFPx7-associated LDs; magenta arrowheads: end-seipin-SNAPf-associated LDs. See Fig 1H for analysis of the data. Numerical values for the graph in (C) can be found in S1 Data. (TIF) [file pbio.3000998.s002.tif]

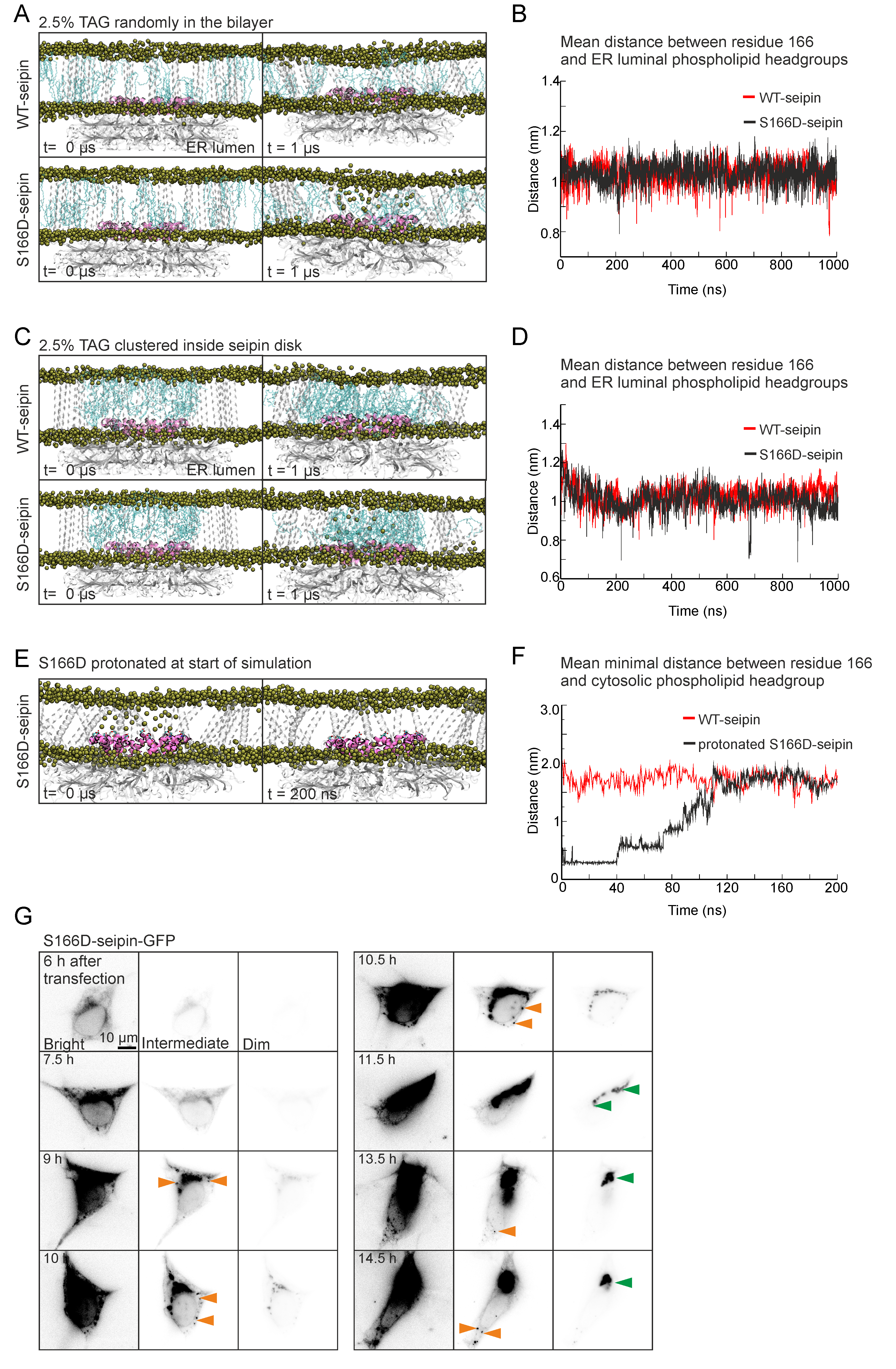

Supplement: S2 Fig — (A) Snapshots of atomistic simulations showing the relative position of α2–α3 helices (pink) with respect to the head group region of the PLs in the bilayer (represented by phosphate atoms shown as yellow spheres) at the beginning (0 μs) and end (1 μs) of simulation. The system consists of 2.5 mol% TAG randomly distributed in the bilayer around the seipin oligomer. The transmembrane helices and luminal domain are shown in white (transparent). The TAGs are shown in cyan (transparent). (B) Center of mass distance between the residue 166 (all) and the phosphate atom of PLs in the luminal leaflet of the bilayer over the simulation period for the systems shown in (A). (C) Snapshots of atomistic simulations showing the relative position of α2–α3 helix with respect to the head group region of the PLs in the bilayer at the beginning (0 μs) and end (1 μs) of simulation. The system consists of 2.5 mol% TAG clustered within the lumen of the seipin oligomer. Coloring as in (A). (D) Center of mass distance between residue 166 (all) and the phosphate atom of PLs in the luminal leaflet of the bilayer over the simulation period for the systems shown in (C). (E) Snapshots of atomistic simulations. Left snapshot shows the membrane deformation (depicted by the invagination of phosphate atoms from the cytosolic leaflet into the bilayer) in the S166D-seipin system. All S166D residues were then protonated (0 μs) and simulated. Right snapshot at the end of the 200-ns simulation shows that the deformation has relaxed. Coloring as in (A). (F) Minimum distance between the protonated S166D residue 166 (all) and phosphate atoms of the PLs in the cytosolic leaflet of the bilayer over a simulation period of 200 ns. Similar data for wild-type S166 residue is shown for reference. (G) HEK 293A cells were transfected with S166D-seipin-GFP and imaged live by widefield microscopy. A single cell is shown over time; orange arrowheads indicate smaller GFP foci, which upon rising fluorescence levels coale [file pbio.3000998.s003.tif]

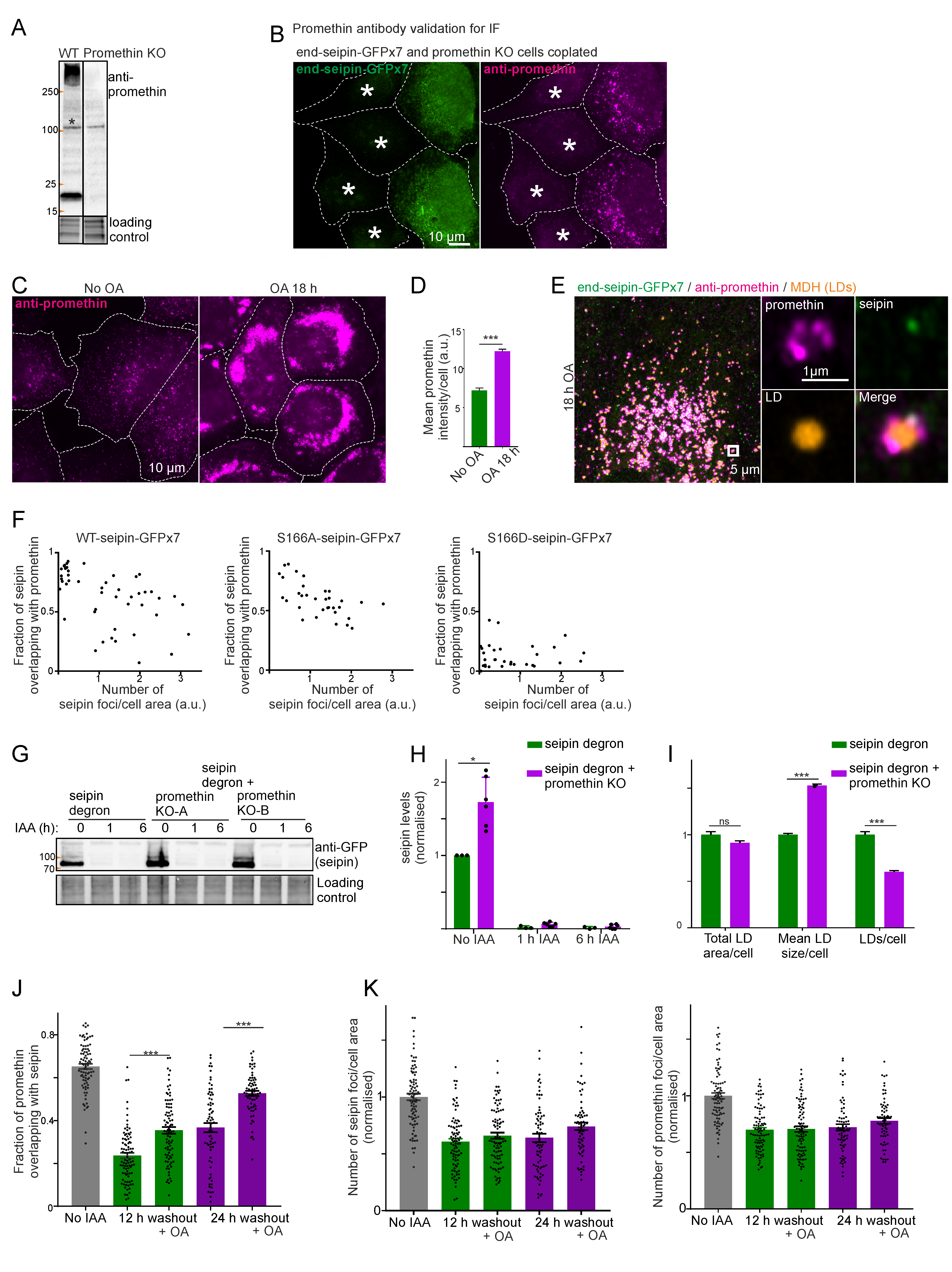

Supplement: S3 Fig — (A) Representative immunoblot of WT A431 cells and promethin KO cell pool using anti-promethin antibody. Asterisk indicates unspecific band. Note both monomer-sized (17 kDa) and larger (>250 kDa) specific bands. (B) End-seipin-GFPx7 cells and promethin KO cells were co-plated, fixed, and stained with anti-promethin antibodies. Asterisks indicate promethin KO cells that have not been engineered to harbor seipin-GFPx7. Note bright promethin staining in the end-seipin-GFPx7 cells. Maximum projections of widefield z-stacks. (C) Cells were cultured in complete medium or additionally treated with 200 μM OA for 18 h, fixed, and stained with anti-promethin antibodies. (D) Analysis of (C). Bars: mean ± SEM, n = 71–180 cells/group, representative experiment repeated once with similar results. Statistics: Mann–Whitney test. (E) End-seipin-GFPx7 cells were treated with 200 μM OA for 18 h, fixed, and stained with anti-promethin antibodies and MDH. Maximum projection of 2 Airyscan z-slices 210 nm apart. (F) In relation to Fig 3B, the fraction of seipin foci overlapping with promethin foci is plotted relative to the number of seipin foci per cell area. A value of 1 in number of seipin foci per cell area corresponds to the mean number of seipin foci per cell area detected in end-seipin-GFPx7 cells in S1C Fig. (G) Immunoblots of seipin degron cells with or without promethin KO treated with IAA. (H) Analysis of (F). Bars: mean ± SEM, n = 3–6 replicates/group, 2 experiments. Promethin KO data are pooled from KO-A and KO-B pools. Statistics: Mann–Whitney test. (I) In relation to Fig 3C and 3D, an additional representation of the “No IAA” data of that panel. Seipin degron cells ± promethin KO were delipidated for 3 d and treated with OA for 1 h as in Fig 3C, and LDs were analyzed. Bars: mean ± SEM, n > 500 cells/group, 3 experiments. Promethin KO data are pooled from KO-A and KO-B pools. Statistics: Mann–Whitney test. (J) Additional analysis of the data in Fig 3I and 3J. Cells were trea [file pbio.3000998.s004.tif]

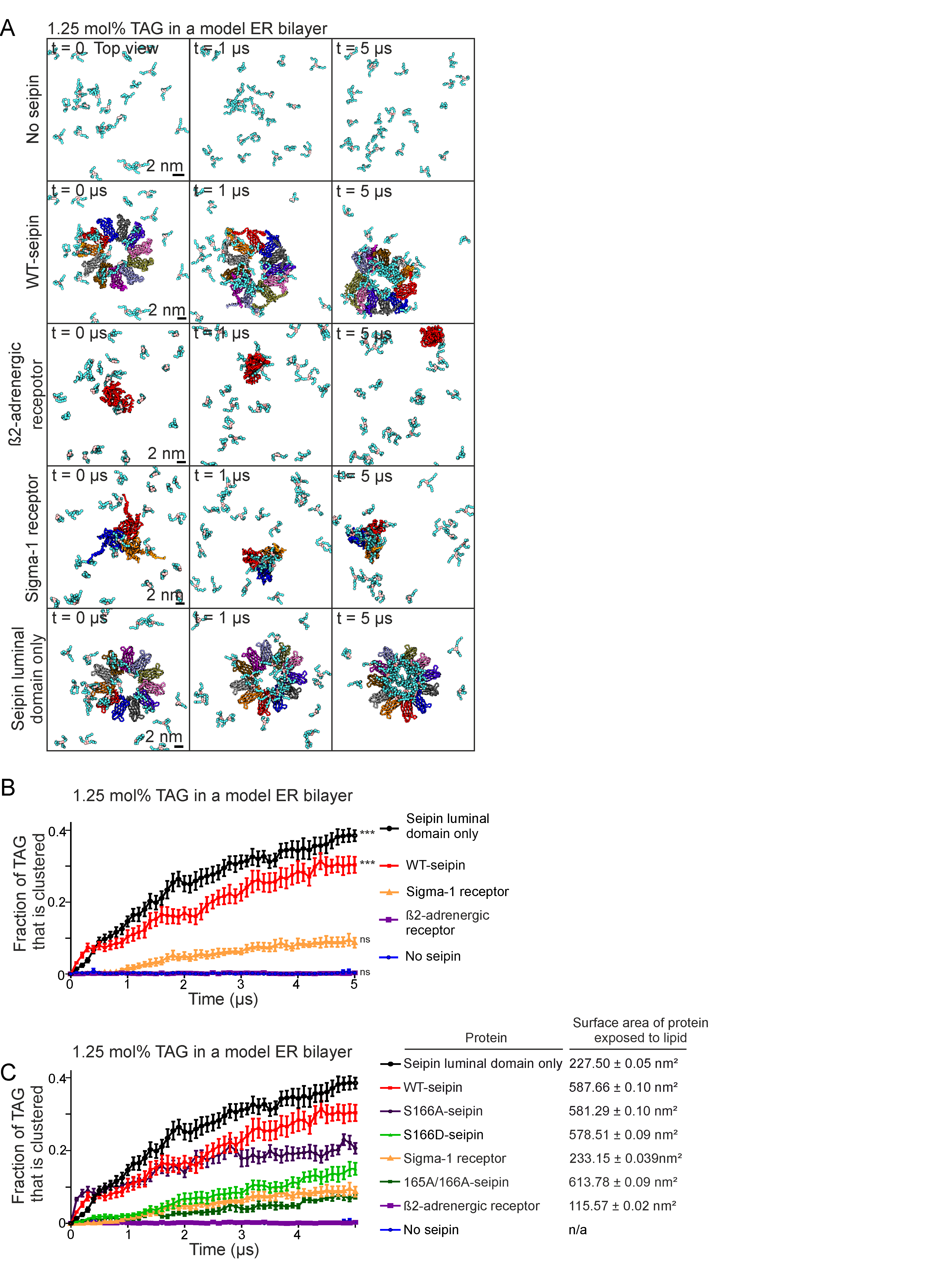

Supplement: S4 Fig — (A) In relation to Fig 4A. Snapshots of coarse-grained simulations with 1.25 mol% TAG in the bilayer. Results are shown for 2 additional integral membrane proteins to demonstrate their TAG affinities. The color coding is as in Fig 1A. Snapshots of “No seipin” and “WT seipin” are the same as in Fig 4A. (B) Analysis of (A). Data points: mean ± SEM, n = 10 simulations/system. Data for “No seipin” and “WT seipin” are same as in Fig 4B. Statistics are based on the final time points of analysis using Kruskal–Wallis test followed by Dunn’s test, comparing against no seipin. (C) Additional analysis of the data in Fig 4B and (B). Total surface area of protein exposed to membrane lipids was calculated using GROMACS tool sasa. Data points: mean ± SEM; data is from 10 simulations/system. Whilst membrane proteins are expected to reduce the mobility of nearby lipids relative to the membrane exposed protein surface area (the so called blocking effect), this does not explain the TAG-clustering effect of WT seipin. Numerical values for the graphs in (A), (B), and (C) can be found in S1 Data. (TIF) [file pbio.3000998.s005.tif]

Uncropped immunoblots

Figure 3G

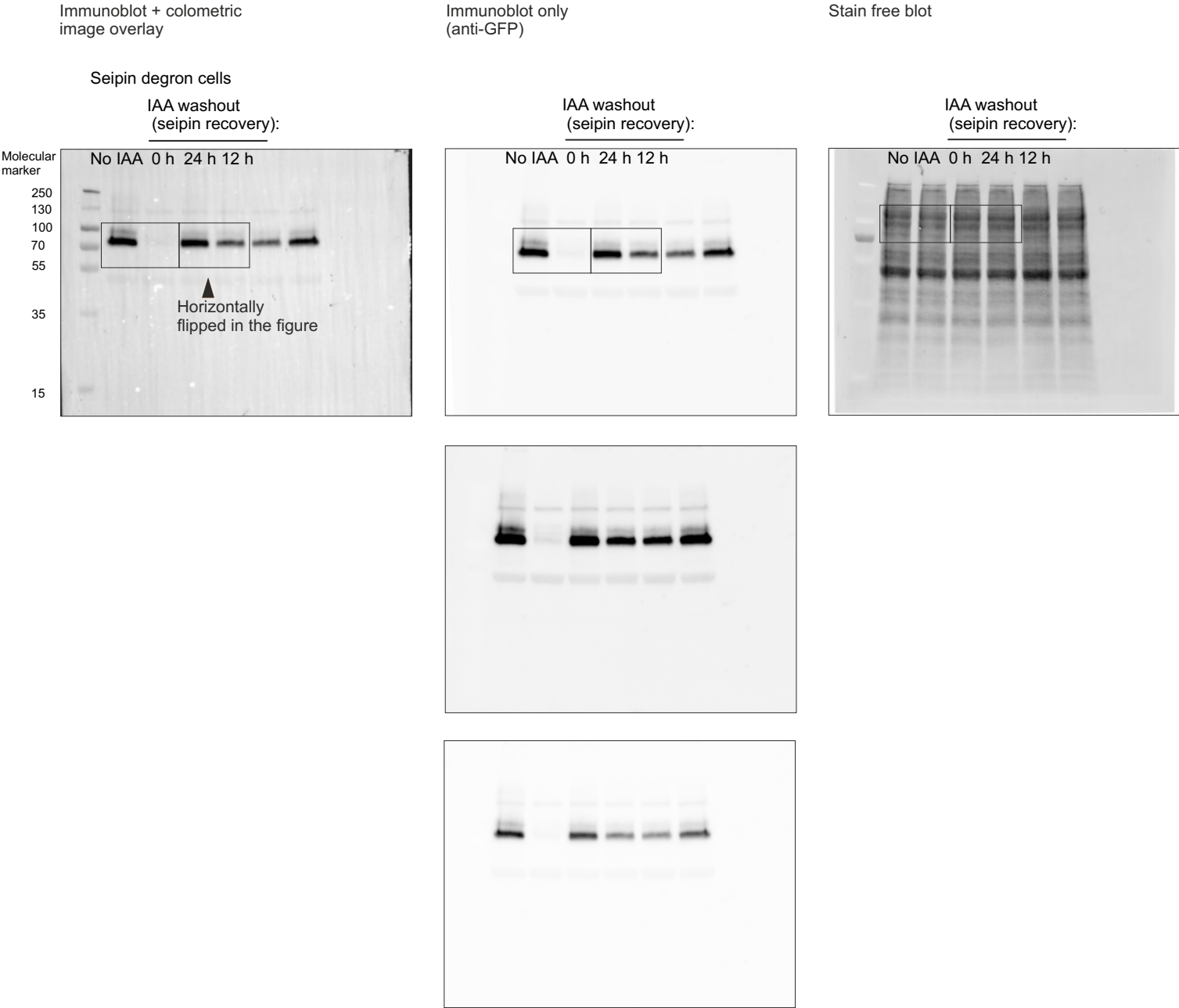

## Figure S3A

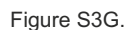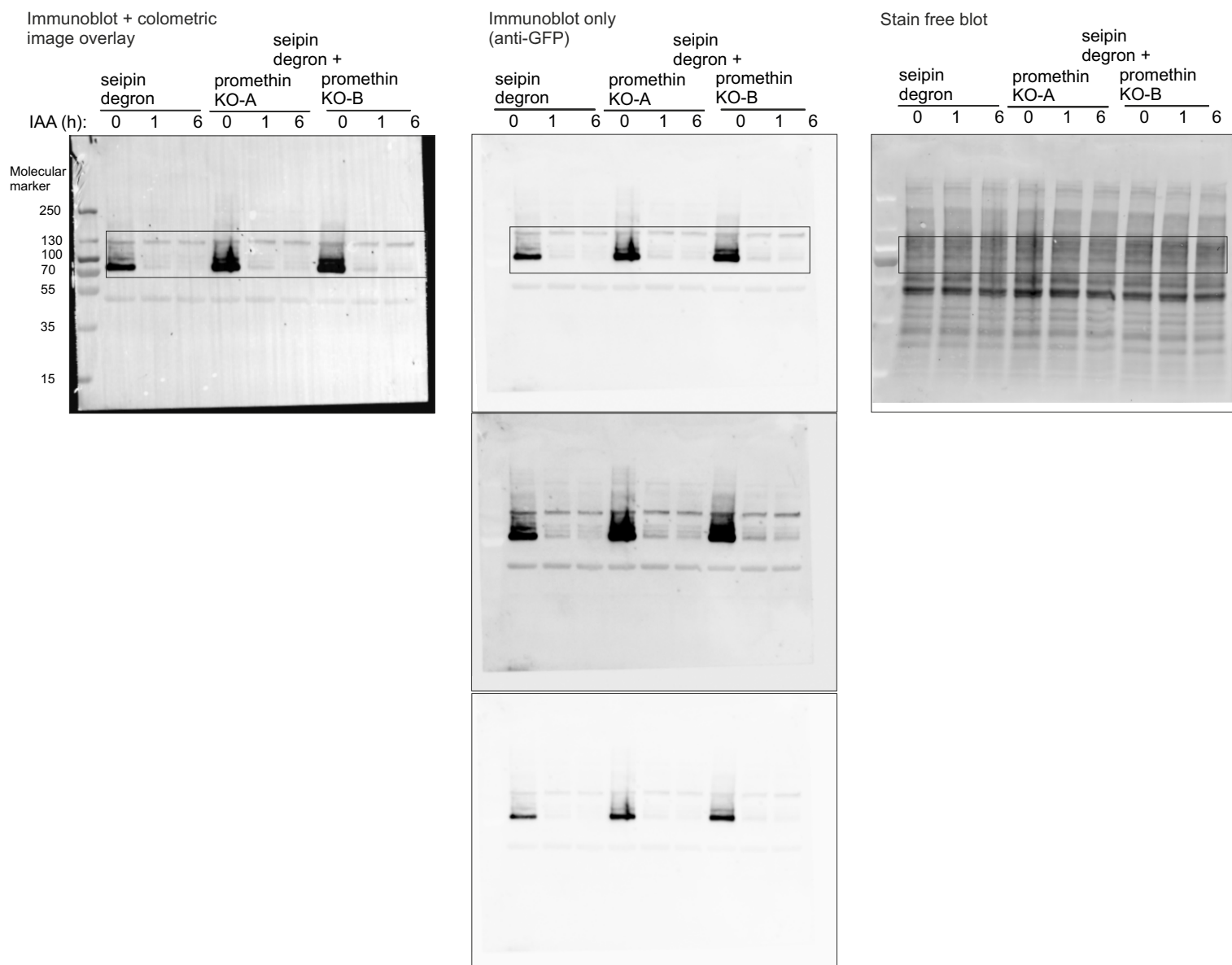

Supplement: S1 Raw Images — (PDF) [file pbio.3000998.s006.pdf]
