# Supplementary material for: Seipin traps triacylglycerols to facilitate their nanoscale clustering in the endoplasmic reticulum membrane
Source: PLoS Biol. 2021 Jan 22;19(1):e3000998. doi: 10.1371/journal.pbio.3000998 (PMC7857593; doi:10.1371/journal.pbio.3000998)
Supplement: S1 Table — The membrane was comprised of 13 different lipid species with molar concentrations identified in the table. (DOCX) [file pbio.3000998.s007.docx]

| **Lipid type** | **Total molar concentration** | |
| --- | --- | --- |
|  | *1.25 mol% TAG* | *2.5 mol% TAG* |
| 16:0-18:1 (∆9-cis) PC | 24.27 | 23.02 |
| 18:1 (∆9-cis) PC | 10.89 | 10.89 |
| 16:0-18:2 (∆9-cis, ∆12-cis) PC | 7.34 | 7.34 |
| 16:0-16:1 (∆9-cis) PC | 6.44 | 6.44 |
| 16:0-18:1 (∆9-cis) PE | 15.03 | 15.03 |
| 18:1 (∆9-cis) PC | 15.03 | 15.03 |
| 16:0-18:1 (∆9-cis) PI | 10.00 | 10.00 |
| 16:0-18:1 (∆9-cis) PS | 3.95 | 3.95 |
| 18:1 (∆9-cis) PC | 2.95 | 2.95 |
| 16:0-18:1 (∆9-cis) PA | 0.95 | 0.95 |
| 18:1 (∆9-cis) PA | 0.95 | 0.95 |
| Cholesterol | 0.95 | 0.95 |
| TAG  (triolein) | 1.25 | 2.50 |
