# Supplementary material for: Seipin traps triacylglycerols to facilitate their nanoscale clustering in the endoplasmic reticulum membrane
Source: PLoS Biol. 2021 Jan 22;19(1):e3000998. doi: 10.1371/journal.pbio.3000998 (PMC7857593; doi:10.1371/journal.pbio.3000998)
Supplement: S2 Table — (DOCX) [file pbio.3000998.s008.docx]

| Simulation | Seipin | Membrane system  (mol% TAG) | Simulation time (in μs) | No. of simulations |
| --- | --- | --- | --- | --- |
| Atomistic | Wild-type | 2.5 (randomly distributed) | ~1 | 2 |
|  | Wild-type | 2.5 (clustered within lumen) | ~1 | 2 |
|  | Mutant (S166D) | 2.5 (randomly distributed) | ~1 | 2 |
|  | Mutant (S166D) | 2.5 (clustered within lumen) | ~1 | 2 |
|  | Mutant (S166D) protonated | 2.5 (randomly distributed) | 0.2 | 2 |
|  | Mutant (S166A) | 2.5 (clustered within lumen) | ~1 | 2 |
|  | Mutant (S165A-S166A) | 2.5 (clustered within lumen) | ~1 | 2 |
| Coarse-grained | No seipin | 2.5 | 5 or 30 | 10 |
|  | Wild-type | 2.5 | 5 or 30 | 10 |
|  | No seipin | 4.85 | 5 | 10 |
|  | Wild-type | 4.85 | 5 | 10 |
|  | Mutant (S166D) | 4.85 | 5 | 10 |
|  | Mutant (S166A) | 4.85 | 5 | 10 |
|  | Mutant (S165A-S166A) | 4.85 | 5 | 10 |
|  | No seipin | 1.25 | 5 | 10 |
|  | Wild-type | 1.25 | 5 | 10 |
|  | Mutant (S166D) | 1.25 | 5 | 10 |
|  | Mutant (S166A) | 1.25 | 5 | 10 |
|  | Mutant (S165A-S166A) | 1.25 | 5 | 10 |
|  | ß2-adrenergic receptor | 1.25 | 5 | 10 |
|  | Sigma-1 receptor | 1.25 | 5 | 10 |
|  | Seipin luminal domain only | 1.25 | 5 | 10 |
